# Supplementary material for: Engineering Skeletal Muscle Grafts with PAX7::GFP-Sorted Human Pluripotent Stem Cell-Derived Myogenic Progenitors on Fibrin Microfiber Bundles for Tissue Regeneration
Source: Bioengineering (Basel). 2022 Nov 15;9(11):693. doi: 10.3390/bioengineering9110693 (PMC9687588; doi:10.3390/bioengineering9110693)
Supplement: Supplementary file 1 [file bioengineering-09-00693-s001.zip › bioengineering-1995363-supplementary.pdf]

bioengineering

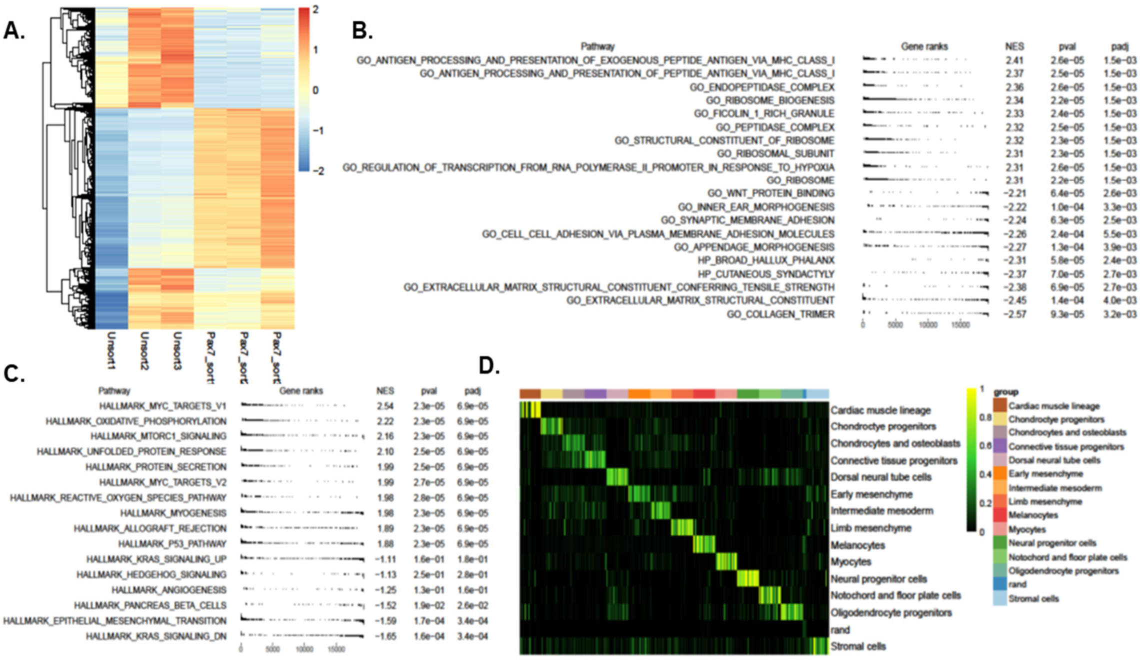

Figure S1. RNA Sequencing supporting Data and CellNet classifier validation. (**A**) Hierarchically clustered heatmap of all significantly differentially expressed genes between sorted and unsorted groups. (**B**,**C**) Gene sets within the C5 Gene Ontology (**B**) and Hallmark (**C**) GSEA sets with highest and lowest normalized enrichment scores representing sets most highly enriched in sorted and unsorted cells respectively. (**D**) Validation of training of the CellNet classifier using held-out data from the mouse organogenesis cell atlas fetal days 9.5-13.5. Held-out cells corresponding to each cell type classifier show a generally high classification score, validating the fidelity of the classification algorithm.
